# Growing Renal Vein Aneurysm Treated by Endovascular Repair: A Case Report and Literature Review

**DOI:** 10.3400/avd.avd.cr.23-00041

**Published:** 2023-11-14

**Authors:** Hisao Nagato, Makoto Wakamiya, Kiyosumi Maeda, Kazuhiko Doi, Hiromasa Kira, Koji Ueyama

**Affiliations:** 1 Department of Cardiovascular Surgery, Nagahama City Hospital, Nagahama, Shiga, Japan; 2 Department of Radiology, Nagahama City Hospital, Nagahama, Shiga, Japan; 3 Department of Radiology, Omi Medical Center, Kusatsu, Shiga, Japan

**Keywords:** renal vein aneurysm, endovascular repair, vascular plug

## Abstract

Renal vein aneurysm (RVA) is extremely rare and often asymptomatic, disclosed only incidentally on diagnostic imaging modalities such as computed tomography and ultrasonography. Management is often just watchful follow-up, but some patients require intervention. We present the case of a 74-year-old man complaining of lower back pain in whom a 53-mm RVA was identified. He underwent successful endovascular repair using Amplatzer vascular plugs. The aneurysm had completely resolved by 12 months. Endovascular treatment of a primary RVA does not seem to have been reported previously. This is a milestone case in the management of RVA.

## Introduction

Primary renal vein aneurysm (RVA) is a very rare entity that is differentiated from venous varices or distention. Seventeen cases have been reported as individual case studies (**Table 1**), and one systematic review from Greece in 2009 described six cases of RVA.^1)^ Some were identified incidentally, while others were identified in patients presenting with characteristics such as pain, pulmonary embolism, or rupture. The latter group underwent surgeries such as vascular reconstruction or nephrectomy. However, no consensus has yet been established regarding the optimal treatment strategy. We present a case of growing giant RVA that was repaired using vascular plugs.

**Table 1:** Reported cases of RVA

Author	Year published	Case profiles					
		Size (cm)	Age (years)	Sex	Location	Presentation	Management
Paschos KA	2019	3.8	58	M	R	Rupture	Nephrectomy
Madden NJ	2019	4.4	37	M	R	Left flank pain, then spontaneously resolved	No intervention
Rios A	2017	4.9	23	F	L	Pulmonary embolism	Open reconstruction
Filis K	2016	7.8	40	F	L	Asymptomatic	No intervention
Yoo J	2016		27	M	L	Asymptomatic	No intervention
Özyüksel A	2016		36	F	L	Pulmonary embolism, pelvic pain	Ex vivo resection and autotransplanted
Zhu et al.^3)^	2015	2.8	44	F	R	Hematuria	No intervention
Dale RT	2014	2.3	57	M	Transplanted	Discomfort	No intervention
Prabakar D	2012	3.9	29	M	L	IVC thrombosis, asymptomatic	No intervention
Rao MV	2011	5	66	M	R		Nephrectomy
Lin TC	2010	3.5	36	F	L	Asymptomatic	No intervention
Ferrante A	2005		73	M	R		Nephrectomy
Yoneyama et al.^2)^	2003	4	57	F	L	Asymptomatic	No intervention
Khader SM	1999		40	M	L	Chronic mid-abdominal pain	
Kabaalioğlu et al.^5)^	1997	5	54	M	L	Abdominal pain	
Krinsky G	1997	5	73	M	R		
Irace L	1994		61	M	L	Asymptomatic	Aneurysmorrhaphy
							
Nagato H	Present case	5.3	74	M	L	Lower back pain	EVT

RVA: renal vein aneurysm; M: male; R: right renal vein; F: female; L: left renal vein; IVC: inferior vena cava; EVT: endovascular treatment

## Case Report

**Figure 1:**
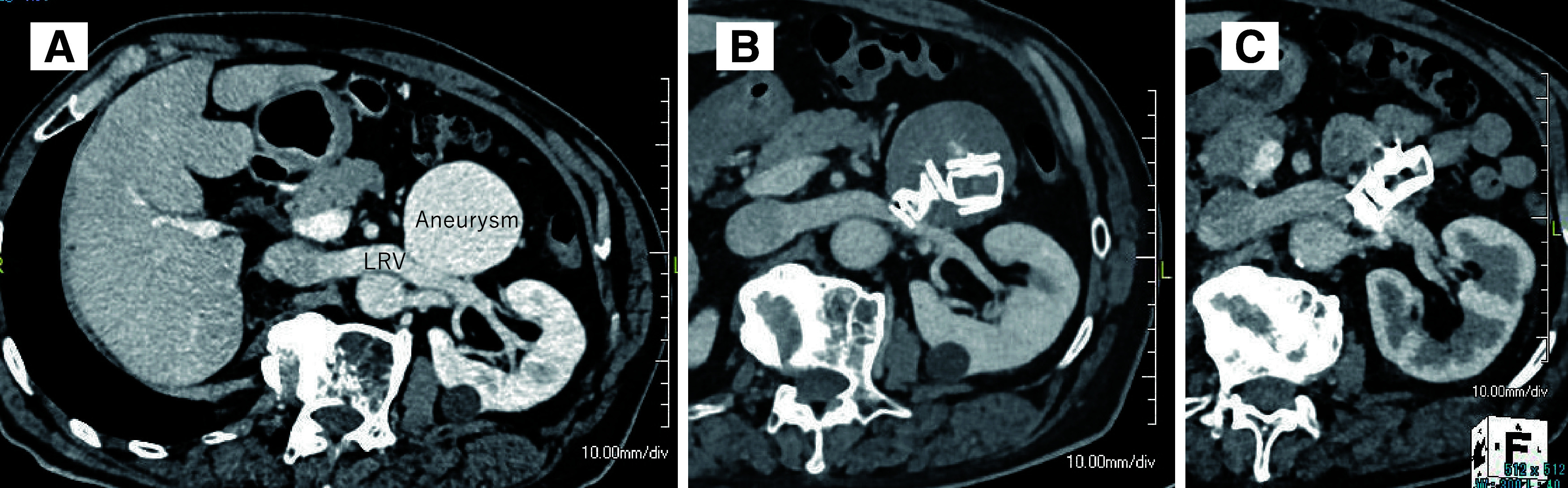
Fig. 1 Serial enhanced CT. (**A**) CT just before the intervention shows a large aneurysm, 53 mm in diameter, deriving from the LRV. (**B**) Immediately after the procedure. The left renal vein is patent. (**C**) One year after the procedure. The aneurysm has disappeared completely. No thrombus is present in the LRV. CT: computed tomography; LRV: left renal vein.

**Figure 2:**
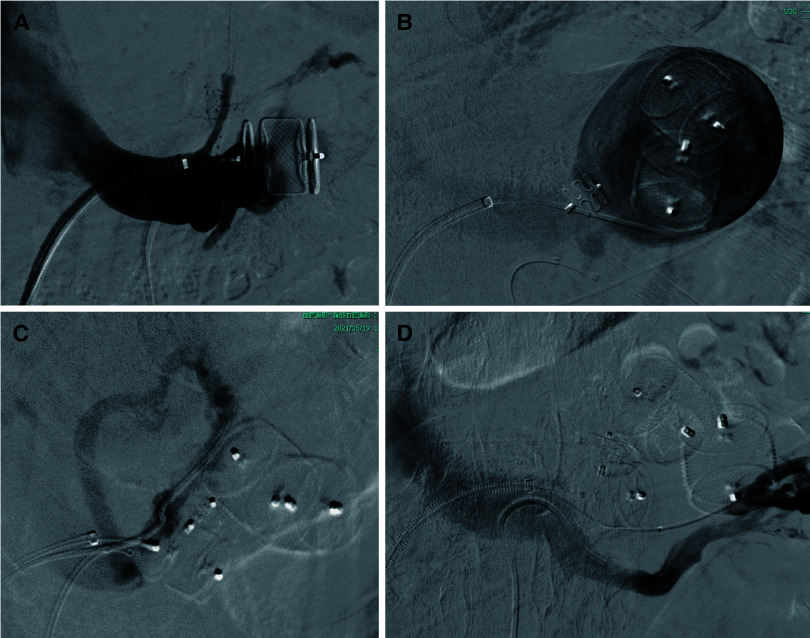
Fig. 2 Angiography during the procedure. (**A**) A 22-mm AVP II in the aneurysm neck obstructs the renal vein. The adrenal vein and the testicular vein are proximal to the aneurysm. (**B**) A 10-mm AVP II cannot close the neck completely. (**C**) A 16-mm AVP II in the neck. Collateral circulation functions for the venous return from the upper branch. (**D**) A 16-mm AVP II in the neck. The venous return from the lower branch is no problem. AVP: Amplatzer vascular plug.

A 74-year-old man visited the emergency department three times in April 2020 due to lower back pain. Computed tomography (CT) disclosed an RVA with a diameter of 48.9 mm. No other potential causes of pain were identified. His medical history included chronic atrial fibrillation, moderate mitral regurgitation, and cerebral infarction. He did not have any history of trauma. Previous CT images that had been obtained for other reasons were reviewed retrospectively and revealed the RVA with a diameter of 17.1 mm in July 2015 and 35.7 mm in September 2019. The aneurysm kept gradually enlarging and had reached 53 mm (**Fig. 1A**), although the lower back pain had almost disappeared. The aneurysm was approached through the right femoral vein. A catheter was also inserted to the left renal artery (LRA). Anatomical information was obtained by LRA-occluded venography, LRA-unoccluded venography, or delayed venous phase imaging of LRA injection. The neck of the aneurysm appeared to be approximately 12 mm on CT and venography, but it was not clearly shown. There was no arteriovenous fistula (AVF). As a preliminary step, we confirmed these things experimentally. When the aneurysm neck was closed with a 22-mm Amplatzer vascular plug (AVP) II (Abbott, Abbott Park, IL, USA) with one lobe of the device positioning in the renal vein and two lobes in the aneurysm, the renal vein itself was completely occluded (**Fig. 2A**). When the neck was closed with a 10-mm AVP II, it did not occlude the renal vein, while the neck was incompletely closed (**Fig. 2B**). Although an additional plug would be required to completely occlude the neck, placing a 10-mm AVP II in the neck first to restrict blood flow appeared safe and effective. Based on this information, we then moved on to the actual procedure. Two 22-mm AVP II were placed within the aneurysm sac to reduce the empty space and promote thrombus formation in the aneurysm lumen. A 10-mm AVP II was delivered in the neck, but after a while, it turned out to be dislodged into the aneurysm. We tried to place a 16-mm AVP II to close the neck. We confirmed that the venous return from the upper branch could flow through collateral circulation (**Fig. 2C**) and the return from the lower branch was not obstructed (**Fig. 2D**). We deployed the 16-mm AVP II. The final angiogram demonstrated complete disappearance of flow into the aneurysmal sac.

The patient’s course following endovascular treatment (EVT) was uneventful. CT just after the procedure still showed the remnant aneurysm sac (**Fig. 1B**). The aneurysm shrank and had completely resolved by 12 months after EVT, without any renal vein thrombosis or obstruction (**Fig. 1C**). The patient has remained on direct oral anticoagulants for atrial fibrillation, and as of nearly 2 years after EVT is doing well without any recurrence.

The patient provided consent for publication with the accompanying images, and the ethics board of Nagahama City Hospital approved this therapy and publication in the journal (No. R5-6).

## Discussion

Primary RVA is a rare disease. A search of PubMed using the three search terms of “renal,” “vein,” and “aneurysm” and the reference sections of the identified articles revealed 17 cases of primary RVA. RVA caused by nutcracker syndrome (NCS) or AVF and pseudoaneurysm of renal vein were excluded. The present case is the 18th case of primary RVA. Profiles of the reported cases, including the present case, are summarized in **Table 1**.

Regarding location, RVA has been said to occur more often on the left side because of the greater developmental complexity of the left renal vein compared to that of the right.^1^^,^^2)^ A review by Zhu et al., however, found no dominant laterality.^3)^ That review included cases of RVA secondary to AVF. Primary RVA should be differentiated from idiopathic venous varices (and solitary varix in particular) or distended veins secondary to NCS or AVF.^2)^ A distended renal vein is a normal variant, showing as a diffusely enlarged vein.^4)^ An idiopathic renal vein varix is usually smaller than an aneurysm, typically not saccular, and accompanied by a dilated venous network.^5)^ Our review of the literature reconfirmed that primary RVA more often occurs in the left renal vein with a left-to-right ratio of 11:6, as shown in **Table 1**.

No consensus has yet been reached regarding treatment strategies. We chose EVT based on the minimal invasiveness of the intervention. To the best of our knowledge, this represents the first case of primary RVA treated using vascular plugs. We used an AVP II to close the aneurysm orifice and three AVP II to achieve sac thrombosis in case of residual passage through the orifice. Successful neck plug occlusion of saccular aneurysm other than RVA has been described in several case reports.^6)^ It is important to close the aneurysm without occluding the renal vein. When closing the aneurysm neck with AVP II, we propose that the device should be placed across the neck with one lobe in the renal vein and two lobes in the aneurysm. We reasoned that this placement would minimize the risk of renal vein occlusion and that little migration of the plug might occur. The 10-mm AVP II placed in the aneurysm neck dislodged into the aneurysm. This may be because the device was not precisely placed across the neck in the intended manner. If accurate placement can be achieved with the method we propose, we believe no further steps need to be taken to increase safety. If not, options such as the proximal balloon occlusion of the renal vein may be appropriate.

We placed plugs within the aneurysm sac to promote thrombus formation and reduce the size of aneurysm. This method has not been reported so far; we believe this is an effective method. Even with modest residual neck blood flow, this approach would reduce the risk of renal vein occlusion by avoiding placement of additional plugs and coils in the neck. AVP is made of braided nitinol mesh and has multi-braided layer structure that enhances thrombogenicity.^7)^

Coil embolization was not considered for this case due to the size of the aneurysm. Reasons include decreasing procedure time, reducing radiation dose, reducing costs, avoiding the risk of coil migration, and avoiding noise shadow on follow-up CT.

We considered treatment of this case with a covered stent would have been risky because of the possibility of dislodgement. Experience with and data about EVT for renal vein lesions remain scarce. These interventions are not designed for the treatment of primary RVA, but articles on EVT for NCS or pseudoaneurysm may be helpful in considering the use of EVT for primary RVA. Two relatively large volume reports have examined stent placement for NCS.^8^^,^^9)^ Stent migration occurred in 2 of 30 cases (6.7%) described by Wang et al.^8)^ and 3 of 60 cases (5.0%) described by Chen et al.^9)^ One of the three cases with stent migration in the report by Chen et al. required open-heart surgery to retrieve a stent from the right atrium. The migration rate cannot be overlooked. A renal vein with NCS even shows definite stenosis at the stent delivery site in general. Such data support our decision not to use a stent in the present case. In another report, a covered stent was placed in the renal vein to treat a pseudoaneurysm caused by blunt trauma from a growing retroperitoneal hematoma.^10)^ That intervention had a life-saving therapeutic purpose. Although the postintervention hospital course was uneventful in that case and the patient was discharged in good condition, no information was provided about the course after hospital discharge. EVT has been commonly used in aneurysm treatment. As a treatment for renal vein lesions, EVT is also a very useful option. However, care should be taken to address concerns such as thrombosis or obstruction of the renal vein, and above all, device migration.

## Conclusion

We successfully repaired a primary RVA using AVP II. We believe that vascular plugs are a useful and safe option in the treatment of RVA. This appears to represent the first description of primary RVA successfully treated endovascularly using vascular plugs.

### Disclosure Statement

The authors have no conflicts of interest to declare.

### Author Contributions

Study conception: HN

Data collection: HN

Analysis: HN, MW, and KD

Manuscript preparation: HN

Critical review and revision: all authors

Final approval of the article: all authors

Accountability for all aspects of the work: all authors.
